# Atopy patch tests are useful to predict oral tolerance in children with gastrointestinal symptoms related to non-IgE-mediated cow's milk allergy

**DOI:** 10.1186/2045-7022-3-S3-P41

**Published:** 2013-07-25

**Authors:** R Nocerino, V Granata, V Pezzella, L Leone, M Di Costanzo, A Passariello, G Terrin, R Troncone, R Berni Canani

**Affiliations:** 1 Department of Pediatrics, University of Naples "Federico II", Italy; 2 Monaldi Hospital, Naples, Italy; 3 Department of Woman's Health and Territorial Medicine, University of Rome “La Sapienza", Rome, Italy; 4 Department of Pediatrics and European Laboratory for the Investigation of Food Induced Diseases (ELFID), University of Naples “Federico II”, Naples, Italy

## Background

Oral food challenge (OFC) is required to establish the persistence or resolution of cow’s milk allergy (CMA). Atopy patch test (APTs) are useful in the initial diagnostic approach in children with non-IgE-mediated CMA. We aim to investigate the benefit of APTs in predicting a reaction to the OFC in children with non-IgE-mediated CMA.

## Methods

We enrolled consecutively children with CMA admitted for OFC to reassess their allergy. The APTs were performed using a drop (20 µl) of fresh cow’s milk (CM) containing 3.5% fat placed on filter paper and applied with adhesive tape to the unaffected skin of the child’s back using a 12-mm aluminum cup. Isotonic saline solution was used as negative control to exclude false positive reactions. The occlusion time was 48 h, and the results were read 20 min and 24 h after removal of the cups. Antihistamines and anti-inflammatory agents were discontinued at least 7 days before the test. All tests were performed by the same nursing staff, and the results were read by two expert pediatric allergists blind to the outcome of OFC. Skin findings were recorded on a standardized form. Reactions were judged to be either negative or positive. Positive skin reactions on the APTs site were classified mild (erythema and slight infiltration, +), moderate (erythema plus papules, ++), or severe (erythema plus vesicles, +++). The OFC was performed after 12 months of exclusion diet. Accuracy of APTs and their correlation (Spearman’s Test) with OFC results were calculated.

## Results

172 children (97 boys, 56.4%; age 6.37 months, range 2–12 months) with CMA-related gastrointestinal symptoms were evaluated. Gastrointestinal symptoms at presentation were vomiting (72, 41.9%), chronic diarrhea (117, 68%), abdominal pain (45, 26.2%). At diagnosis 113/172 (65.7%) children had positive APTs to cow’s milk proteins (CMP). After 12 months of exclusion diet 94 children outgrown CMA. The APTs performed immediately before OFC at 12 months showed a sensitivity of 67.95% (95%CI 56.42-78.07), specificity of 88.3% (95%CI 80.03-94.01), PPV of 82.81% (95%CI 71.32-91.1), NPV of 76.85% (95%CI 67.75-84.43) and a LR of 5.80 (95%CI 3.35-10.38). APTs were significantly correlated (p<0.001) with OFC outcomes (r 0.579).

## Conclusion

The APTs are a valuable tool in the follow-up of pediatric patients with non-IgE-mediated CMA by contributing in determining whether an oral challenge can safely be undertaken.

## Disclosure of interest

None declared.

